# Pathway Analysis of Selected Circulating miRNAs in Plasma of Breast Cancer Patients: A Preliminary Study

**DOI:** 10.3390/ijms21197288

**Published:** 2020-10-02

**Authors:** Veronika Holubekova, Zuzana Kolkova, Marian Grendar, Dusan Brany, Dana Dvorska, Igor Stastny, Marianna Jagelkova, Katarina Zelinova, Marek Samec, Alena Liskova, Zuzana Laucekova, Erik Kudela, Martina Bobrovska, Michal Kalman, Pavol Zubor, Zuzana Dankova

**Affiliations:** 1 Biomedical Centre Martin, Jessenius Faculty of Medicine, Comenius University in Bratislava, 03601 Martin, Slovakia; zuzana.snahnicanova@uniba.sk (Z.K.); marian.grendar@uniba.sk (M.G.); dusan.brany@uniba.sk (D.B.); dana.dvorska@uniba.sk (D.D.); igor.stastny@gmail.com (I.S.); marianna.jagelkova@uniba.sk (M.J.); katarina.zelinova@uniba.sk (K.Z.); marek.samec@uniba.sk (M.S.); alenka.liskova@gmail.com (A.L.); zuzana.dankova@uniba.sk (Z.D.); 2 Department of Obstetrics and Gynaecology, Jessenius Faculty of Medicine, Comenius University in Bratislava, Martin University Hospital, 03659 Martin, Slovakia; laucekovazuzana@gmail.com (Z.L.); erik.kudela@uniba.sk (E.K.); 3 Department of Medical Biology, Jessenius Faculty of Medicine, Comenius University in Bratislava, 03601 Martin, Slovakia; 4 Department of Pathological Anatomy, Jessenius Faculty of Medicine, Comenius University in Bratislava, Martin University Hospital, 03659 Martin, Slovakia; bobrovska@mfn.sk (M.B.); kalman@mfn.sk (M.K.); 5 Department of Gynaecologic Oncology, The Norwegian Radium Hospital, Oslo University Hospital, 0379 Oslo, Norway; prof.pavol.zubor@gmail.com; 6 OBGY Health & Care, Ltd., 01001 Zilina, Slovakia

**Keywords:** breast cancer, circulating miRNA expression, plasma, miR-99a, miR-130a, miR-484, miR-1260a, normalization, delta–delta Ct

## Abstract

MicroRNAs in the circulation of breast cancer (BC) patients have great potential for the early diagnosis, treatment and monitoring of breast cancer. The aim of this preliminary study was to obtain the expression profile of selected miRNAs in the plasma of BC patients that could discriminate BC patients from healthy volunteers and may be useful in early detection of BC. Significantly deregulated miRNAs were evaluated by pathway analysis with the prediction of potential miRNA targets. The study enrolled plasma samples from 65 BC patients and 34 healthy volunteers. Selected miRNAs were screened in pilot testing by the real-time PCR (qPCR) method, and the most appropriate reference genes were selected for normalisation by the geNorm algorithm. In the final testing, we detected miR-99a, miR-130a, miR-484 and miR-1260a (*p* < 0.05) as significantly up-regulated in the plasma of BC patients. Kyoto Encyclopaedia of Genes and Genomes (KEGG) pathway analysis revealed that all significantly deregulated miRNAs are involved in the Hippo and Transforming Growth Factor-beta (TGF-beta) signalling pathways. Our study confirmed a different profile of selected circulating miRNAs in the plasma of BC patients with an emphasis on some critical points in the analysis process.

## 1. Introduction

Breast cancer is the leading gynaecologic cancer disease worldwide with an estimated incidence of 46.3 age-standardised rate (ASR) and estimated mortality of 13.0 ASR per 100,000 women [1]. In 28 countries of the European Union (EU), a total of 404,920 new BC cases were estimated in 2018, which represents 29.2% of all cancers in women in the EU. A higher incidence of BC is seen in developed countries [2], which according to one theory, could be associated with higher socioeconomic status and better availability and accuracy of screening examinations (mammography, ultrasound examination and magnetic resonance imaging) and life expectancy, as the risk for BC increases after 50 years of life [3].

Breast cancer is heterogeneous disease comprising phenotypically diverse tumours categorised into molecular subtypes based on the overexpression of receptors for the hormones oestrogen (ER) or progesterone (PR), or for the human epidermal growth factor receptor 2 (HER2). According to the St. Galen Consensus 2011, the molecular subtypes of BC are identified as luminal A (ER+/PR+/HER2-/low Ki-67), luminal B (ER+/PR+/HER2-/+/high Ki-67), HER2-overexpression (ER-/PR-/HER2+) and triple-negative/TNBC (ER-/PR-/HER2-). Molecular subtypes of BC are important in the definition of therapy indications, as systematic treatment recommendations, risk and predictive factors should be considered [4].

It is known that hereditary breast cancer represents 5–10% of all BC cases [5]. Other cases are accompanied with risk factors such as early age at menarche and later age at menopause, late age at first birth, nulliparity and fewer children, oral contraceptives and hormone therapy, alcohol consumption, greater BMI and body fat distribution. Physical activity and breastfeeding have a protective effect on the occurrence of BC [6,7]. However, the dual effect of pregnancy and breastfeeding after 35 years may increase the risk of BC [8].

Early detection of BC and appropriate adjuvant therapy contribute to reducing the number of deaths from BC. Currently, many states have established a national screening program of BC, which includes breast self-examination and clinical examination and a gold standard imaging method such as mammography at age-defined intervals [9]. However, mammography is not suitable for pregnant women; moreover, in women younger than 40 years, the dense breast parenchyma can produce false positive and false negative results. A modern procedure currently in the research phase involves the detection of candidate cancer-specific biomarkers non-invasively, in patients’ body fluids. One such method is the detection of microRNAs in the patient’s circulation and this seems to be promising in the improvement of BC screening and in the treatment and monitoring of the disease [10].

MicroRNAs (miRNAs) are a class of short non-coding RNAs 21–23 ribonucleotides in length, with the ability to control protein translation and/or influence the stability of messenger RNA (mRNA). MicroRNAs are key regulators involved in the homeostasis of multiple biological systems such as organ function as well as pathobiological processes, for example cancer and aging [11]. It is still unclear if miRNAs are secreted passively or actively by cells, or if cell-free miRNAs in the circulation are specific or random because of their expression heterogeneity between cancer types, tumour stage, treatment outcome and survival [12].

The aim of this preliminary study was to detect miRNA expression in the plasma of breast cancer patients in order to discriminate BC patients from healthy volunteers; these miRNAs could be useful in the early detection of BC. To control for the role of significantly deregulated miRNAs in the circulation of BC patients, we performed pathway analysis with the prediction of miRNA targets and gene ontology annotations.

## 2. Results

### 2.1. Patient Characteristics

A total of 99 women of the Caucasian race were recruited in the study, including 65 patients with breast cancer (BC) and 34 normal (control) patients. The patient characteristics are summarised in Table 1. The mean age was 56 years (interquartile range IQR; 47, 68) in BC patients and 41 years (IQR; 28, 46.5) in control patients. Of the BC patients, 40% (21/65) presented with ductal cancer, 15% (8/65) with lobular invasive cancer and 45% (24/65) with invasive cancer with no specific type. The composition based on the molecular subtypes of BC was as follows: 63% (33/65) patients were classified as luminal A, 25% (13/65) patients were luminal B, 7.7% (4/65) patients were HER2 positive and 3.8% (2/65) were triple-negative cancers (TNC). The cohort of BC patients was composed of 14% (8/65) grade 1, 53% (30/65) grade 2 and 33% (19/65) grade 3.

### 2.2. MicroRNA Profiling in Plasma of Normal and Breast Cancer Patients

**Figure 1:**
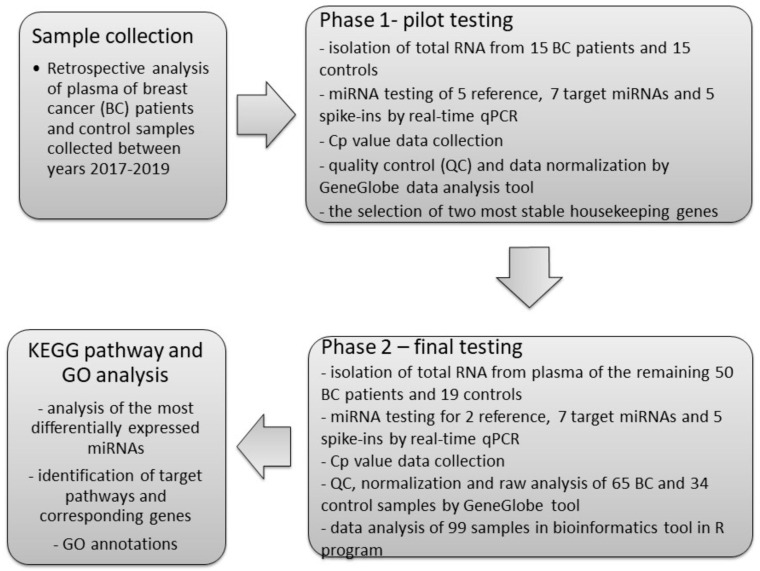
The scheme of study design.

Based on our literature review [10] and our yet unpublished data on breast cancer tissues and circulating miRNA expression, five references [13,14,15,16] and seven target miRNAs [17,18] detectable in plasma were chosen to examine as the most representative biomarkers in plasma of BC patients and control subjects. The process of miRNA profiling consisted of two phases: pilot testing (Phase 1) and final testing (Phase 2). The scheme of the study design is displayed in Figure 1. The quality of total RNA extraction and reverse transcription was checked using synthetic spike-ins. Overall, 65 patients with breast cancer and 34 healthy control subjects without any history of malignancy were tested in both experiments.

#### 2.2.1. Phase 1: Pilot Testing of miRNA Biomarkers

MicroRNA biomarkers were chosen based on a panel of 179 miRNAs detectable in plasma in Exiqon’s database built on total RNA extracted from human blood plasma or serum. Pilot testing was performed on 15 BC and 15 control samples, each sample was analysed in triplicate by miRCURY Locked Nucleic Acid (LNA) miRNA assays using a LightCycler 480 (Roche) and the second derivative method for cycle quantification value (Cq) calculation. Secondary data analysis was performed in the GeneGlobe data analysis tool with the geNorm normalisation method [19] on pre-defined reference miRNAs only. From five analysed miRNAs, miR-520d-5p and miR-1228-3p had no measured expression in plasma samples. The remaining three reference miRNAs had a good stability factors (miR-16-5p–0.108; miR-103a-3p–0.189, miR-191-5p–0.168); nevertheless, the Cq arithmetic mean of miR-191-5p expression in the control and BC groups was higher than 30 cycles and was not suitable as a reference gene. The Cq data were compared to miR-16-5p and miR-103a-3p that were chosen as feasible reference genes for the study. Pilot testing revealed that miR-484 was significantly up-regulated in BC samples (p<0.05) when compared to controls (Table 2).

#### 2.2.2. Phase 2: Final Testing

Profiling of the selected miRNAs was performed in the remaining samples. In the final testing by the GeneGlobe data analysis tool, all BC (n = 65) and control (n = 34) samples were analysed by the same methodology as in pilot testing. The miRNAs miR-191-5p, miR-520d-5p and miR-1228-3p were excluded from the analysis. The average arithmetic means of miR-16-5p (stability factor 0.1164) and miR-103a-3p (stability factor 0.21) was 25.63 and 25.29 in the control and cancer groups, respectively. The final testing confirmed up- or downregulation in seven miRNAs with no significant differences in the plasma of BC patients (Table 2).

### 2.3. Identification of Deregulated miRNA by the ddCt Method

Input data from the GeneGlobe data analysis tool were computed using the standard delta–delta Ct method (ddCt) with miR-16-5p and miR-103a-3p as references. Fold change was calculated, and the homogeneity of data was visualised. Up- or downregulation of miRNAs was confirmed in 100% of analysed samples when compared to the GeneGlobe final experiment (Table 2). The delta–delta Ct method identified miR-99a-5p, miR-130a-3p, miR-484 and miR-1260a as significantly up-regulated in BC patients when compared to control samples.

The causality of all miRNAs and histopathological parameters was tested by robust ANOVA. However, no significant associations were found. The table with *p*-values and dotplots with overlaid medians in cases with weak significance are listed in Table S1.

### 2.4. Signal and Functional Pathway Analysis

The most differentially expressed miRNAs were analysed by KEGG pathway analysis using the DIANA-mirPath v3.0 online web analysis tool to investigate the mechanism of their function and to analyse their target genes. All four miRNAs were significantly enriched in pathways involving the Hippo signalling pathway (hsa04390) and TGF-beta signalling pathway (hsa04350).

MicroRNAs miR-130a-3p, miR-484 and miR-1260a were enriched in the phosphatidylinositol signalling system (hsa04070) and oestrogen signalling pathway (hsa04915). MicroRNAs miR-130a-3p, miR-484 and miR-99a were enriched in the glioma (hsa05214) and mammalian target of rapamycin (mTOR) signalling pathway (hsa04150). The genes affected by the deregulation of miRNAs are presented in Table 3.

**Figure 2:**
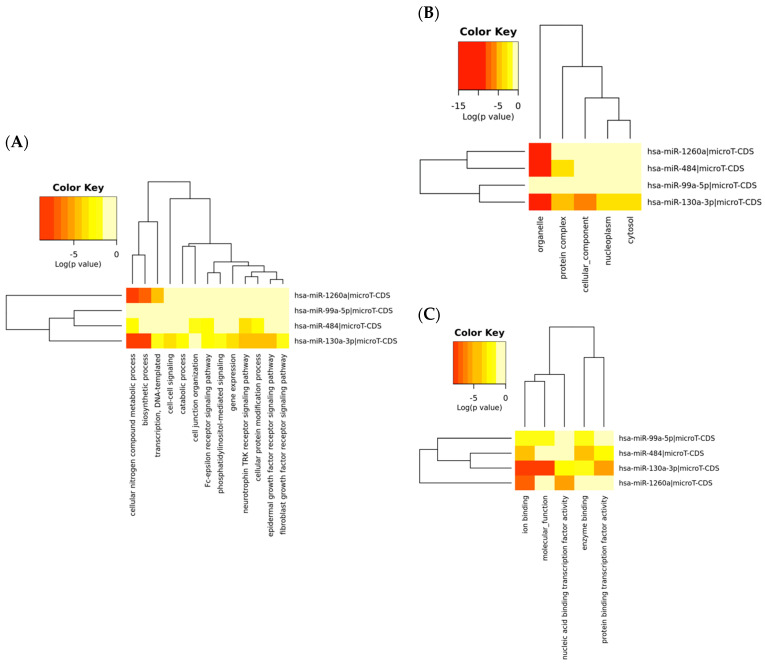
Heatmaps of the Gene Ontology (GO) analysis. GO enrichment of putative targets was performed for four significantly up-regulated miRNAs at three levels: (**A**) biological process, (**B**) cellular component and (**C**) molecular function. Different colours represent different levels of pathway term enrichment in the miRNA target genes. Red indicates a higher degree of enrichment.

The Gene Ontology (GO) analysis was useful to investigate biological function in subcategories such as biological process, cellular component and molecular function corresponding to the target genes regulated by the up-regulated miRNAs. The GO annotation results for the gene union of the target genes of up-regulated miRNAs in breast cancer are listed in Table 4. A more detailed description of the GO categories and corresponding miRNAs is displayed in the heatmap (Figure 2).

## 3. Discussion

The preliminary study targeted differentially expressed miRNAs in the circulation of breast cancer patients compared to healthy volunteers. Breast cancer is a heterogeneous disease with different cell populations within the same tumour [20], and a better understanding is needed regarding breast cancer molecular pathways leading to the development of new therapeutic strategies that have an advantage in the prognosis and diagnosis of BC. A novel approach is based on the fact that tumour cells and/or tumour nucleic acids including miRNAs are released into the circulation and might serve as potential diagnostic markers for tumour development, metastasis and response to treatment [21].

In the technical part of the study, we wanted to overcome some critical points in the steps such as RNA extraction from plasma, reverse transcription, real-time PCR (qPCR) and data analysis. The process of RNA extraction and reverse transcription was controlled by synthetic spike-ins with different concentrations to monitor the presence of inhibitors and sample quality. In qPCR, we used the second derivative method for Cq calculation that has been described as being more reliable and appropriate when a large number of assays are analysed on multiple plates [22]. This procedure allowed us to obtain uniform Cq values without marked variation that could bias the statistical analysis.

We analysed five potentially reference miRNAs that have been described as the most useful in the normalisation of qPCR data. Therefore, the geNorm algorithm in pilot testing was used to find the most stable reference miRNAs with Cq below 30 cycles. Normalisation to more than one reference gene produces more reliable results and reduces the bias due to the differences between samples in the efficiency of miRNA extraction as well as in reverse transcription and amplification. Inadequate choice of reference genes also produces misleading results [23].

In the selection of the most appropriate reference genes, miR-520d-5p and miR-1228-3p were recommended as the most stable and useful in the spectrum of clinical material, such as exosomes, plasma and tissues [24]. However, the study revealed no expression of the mentioned genes in the plasma of either healthy or cancer patients. MicroRNA-191-5p was also excluded after pilot testing because control and BC patients had a Cq higher than 30 cycles; under these conditions, the variability in the amount of miRNA increases and the results may not be representative [25]. The most stable reference miRNAs (miR-16-5p and miR-103a-3p) were also verified in the final testing of control and BC patient plasma. The difference between the control and BC groups was 0.34 cycles in the arithmetic mean of both reference miRNAs. Another approach allows for normalisation to non-human miRNA, using an external control like cel-miR-39, or other small nuclear RNAs longer than 100 nucleotides. However, adding external control hardly balances the amount among samples, and longer RNAs are secreted and protected in the circulation differently to miRNAs [12].

MicroRNA-16 and miR-103a-3p are generally used as references, although some studies have reported a significant downregulation of miR-16-5p in the plasma and/or serum of BC as well as TNBC patients [26]. Ng et al. (2013) do not support the use of miR-16-5p for normalisation because of its elevated expression in the plasma of breast cancer patients [27]. In another study, miR-16-5p was identified as the most consistent reference miRNA between different subtypes of BC tissue [28] and in the plasma of cancer patients [29]. In a study by Chan et al. (2013), the authors identified miR-103a and miR-191 as the most stably expressed miRNAs in the serum of BC patients [30], although serum was identified to contain a higher concentration of miRNA [31]. Increased expression miR-103a-3p together with miR-320a, miR-361-5p and miR-21-5p, were chosen through the Cancer Genome Atlas as the most clinically relevant miRNAs [32]. High expression of the miR-103/107 family is associated with metastasis and a poor prognosis. Induction of epithelial to mesenchymal transition (EMT) via the downregulation of miR-200 has also been reported to be associated with the miR-103/107 family at the cellular level [33]. On the contrary, miR-103 and miR-107 were used as references in a study on the pathologic response to preoperative chemotherapy in TNBC core biopsies [34]. Serum expression of miR-24 and miR-103a was identified as a valuable biomarker for distinguishing atypical hyperplasia and early-stage of breast cancer [35]. Conversely, Normfinder and the geNorm algorithm have found stable expression of miR-103a together with other miRNAs in multiple diseases, e.g., in patients with Parkinson’s disease [36] and renal cell carcinoma [37]. In this study, we identified four significantly up-regulated (miR-99a, miR-130a, miR-484 and miR-1260a) miRNAs in the plasma of breast cancer patients.

MicroRNA-99a has a tumour suppressive role in the mammalian target of rapamycin (mTOR) signalling pathway [38]. It is downregulated in human cancers, which leads to the upregulation of mTOR and insulin-like growth factor receptor type 1 (IGF1R) in cancer tissue [39]. Upregulation of mTOR and fibroblast growth factor receptor 3 (FGFR3) inhibits tumour growth activated by tyrosine-protein kinase cellular sarcoma (c-SRC) [40]. The overexpression of miR-99a inhibits BC cell proliferation, migration and invasion in vivo by targeting FGFR3 and thus might have potential as a therapeutic target [41,42]. mTOR belongs to the PhosphoInositide 3-Kinase (PI3K)-related kinase family (PIKK), and miR-99a has a role in the PI3K/ protein kinase B (Akt) signalling pathway in endometrial cancer through a dual inhibitory effect. Therefore, miR-99a might have potential as an miR-99a mimic in cancer therapy [43]. A study on data of breast cancer tissues and serum samples that validated findings in clinical samples identified mir-99a together with miR-21-3p and miR-21-5p as the most eligible biomarkers for the early identification of breast cancer [44]

MicroRNA-130a expression is aberrant in various types of cancer, including breast cancer, mirroring its different roles as an oncogene or tumour suppressor. Studies on cell cultures and mice showed that miR-130a-3p acts as an onco-miR targeting Phosphatase and Tensin homolog (PTEN) and driving malignant cell survival and growth in the tumour [45]. Another study identifying the relationship between miR-130a and PTEN expression showed that downregulation of PTEN activates the PI3K/Akt/ endothelial Nitric Oxide SynthaseeNOS signalling pathway in the inflammatory response after human coronary artery endothelial cell (HCAEC) injury [46]. Another way of driving cell proliferation and migration has been described through the overexpression miR-130a and suppressing TGFBR2 expression in gastric cancer. Thus, miR-130a expression directly influences the TGF-β signalling pathway and is linked to EMT markers [47]. Plasma expression of miR-130a in BC patients varies according to HER2 status and lymph node positivity [48] and together with cluster miR-17-92, miR-22 and miR-29a/c can differentiate between TNBC and luminal A [49]. On the contrary, exosomal miR-130a-3p expression has a suppressive effect on breast cancer stem cell proliferation, invasion and migration via the downregulation of Ras superfamily of small GTPases-RAB5B; decreased miR-130a-3p expression is associated with lymph node metastasis and advanced tumor stage [50]. MiR-130a is involved in multiple signalling pathways regulating drug susceptibility [51], and studies have shown a heterogeneous pattern of miR-130a leverage.

We identified miR-484 as significantly upregulated (fold change (FC) = 1.22, *p* = 0.0002) in the plasma of BC patients. Similarly, Zaero et al. (2014) found higher expression of miR-484 in the serum (fold change–FC = 1.6, *p* = 0.0026) of early breast cancer patients. In accordance with these authors, we did not find any correlation between histopathological parameters and miR-484 expression [52]. In a study on ovarian cancer cells, the authors ascertained that miR-484, miR-642 and miR-217 are able to predict chemoresistance in ovarian cancer. Analysis of miR-484 revealed that it is involved in the regulation of angiogenic factors and its overexpression downregulates VEGFB and VEGFR2 expression; different levels of miR-484 may reflect the response to therapy [53]. High expression of miR-484 in the serum of patients with non-small cell lung cancer (NSCLC) was positively correlated with histological grade, lymph node metastasis, distant metastasis, clinical stage and poor overall survival [54]. In contrast, miR-484 is recommended as a suitable endogenous control for miRNA experiments [14,55].

Little is known about miR-1260a expression. Some studies have identified miR-1260a as an immune-related miRNA that has an overlapping seed sequence with miR-1260b. Robust miR-1260a/1260b expression in maternal breast milk may impart immune protection on the infant gut [56]. Expression of miR-1260a has been described as bring downregulated in TNBC tissue [57] and high levels of miR-1260a in the serum of metastatic BC patients is associated with a poor prognosis [18].

While the expression of miR-130a and miR-484 was in harmony with published results, miR-99a and miR-1260a were detected in the opposite pattern in the circulation. This discrepant expression is difficult to explain at this time; it could be due to the multiple origins of circulating miRNAs that do not originate only in the tumour environment [58]. It is also difficult to draw a clear conclusion from studies that have examined the expression of circulating miRNAs due to the different methods used to determine expression, i.e., microarray, next generation sequencing or qPCR. There are also differences regarding the qPCR system and what type of chemistry was used (TaqMan probes or SYBRgreen and a melt curve). Another critical point is the data analysis and algorithm to obtain the Cq value. The selection of the most suitable reference miRNA affects the subsequent analysis, and the most suitable references in one study may not be applicable in other studies. Consequently, the results of some studies are incomparable and is very difficult to come to a conclusion regarding these critical points since no precise guidelines for standardisation are available.

In our study, we identified some miRNAs (miR-99a, miR-130a, miR-484 and miR-1260a) that were significantly up-regulated in the plasma of breast cancer patients. At first glance, the fold change was not distinct from the control group. However, the input data are relatively uniform, as we followed several recommendations [23,25,59]. The most important differentially expressed miRNAs were analysed for pathways where they are significantly involved in the development of breast cancer, whether binding sites on target genes are experimentally confirmed or predicted via algorithms. The verification of true miRNA binding to specific target genes was not possible in this study.

A preliminary study has limitations, e.g., small sample size included in the BC and control cohorts and the small subgroups in the evaluation of histopathological parameters and miRNA expression. In subsequent studies, we plan to analyse the miRNA profile of BC tissues and to examine the entire image of circulating miRNAs in the plasma of BC and control cohort by the microarray method.

In subsequent studies, it will be necessary to evaluate circulating miRNA expression in more samples with a standardised and accepted methodology to obtain a broader view on the complex state of BC patients. Accurate information on miRNA expression in the plasma of cancer patients may help with a personalised approach and lead to the development of novel targeted therapy strategies. The level of circulating miRNA expression is further influenced by many factors that are related to the physiological state of the organism, and may even change during the day. The simple idea of early detection of miRNAs in a patient’s circulation then becomes a complex task in which many difficulties have to be overcome.

## 4. Materials and Methods

### 4.1. Patient Recruitment, Blood Collection and Plasma Preparation

All breast cancer patients were recruited at the Clinic of Obstetrics and Gynaecology, University Hospital Martin, Jessenius Faculty of Medicine, Comenius University in Martin and signed the informed consent form for inclusion into the study. The study was approved by the institutional Ethics Committee in accordance with the Declaration of Helsinki. The cohort included 65 breast cancer patients and 34 healthy volunteers. Breast cancer (BC) patients were selected according to the following criteria: (a) all patients were female; (b) all patients were diagnosed with a defined clinical stage; (c) disease was confirmed by routine histopathology; (d) none of the patients had another cancer or disease that may affect the miRNA plasma profile and e) none of the patients underwent preoperative radiotherapy or adjuvant chemotherapy. The healthy cohort was predominantly composed of female faculty staff and female volunteers who wanted to participate in the study with no history of malignant disease and no inflammatory conditions. All blood samples were collected in EthyleneDiamineTetraAcetic acid (EDTA)-anticoagulant tubes and processed as soon as possible, no later than 4 h after collection. The short-term storage and transport temperature was 4 °C. Plasma was separated from blood using two-step centrifugation (3000 revolution per minute/RPM for 10 min and 14,000 RPM for 10 min to minimise or remove the cell debris, residual platelets, microparticles, etc.). The level of haemolysis in plasma samples was evaluated by visual inspection and the measurement of oxyhaemoglobin absorbance at 414 nm. Clear plasma was stored at −80 °C until analysis.

### 4.2. RNA Extraction, Reverse Transcription of RNA and Quality Control

Circulating RNA was extracted from 200 μL of plasma using the miRNeasy Serum/Plasma Advanced kit (Qiagen, Hilden, Germany) according to the manufacturer’s recommended protocol. Total circulating RNA, including the miRNA fraction, was resuspended in 20 μL of RNase-free water. One microliter of total RNA was then reversely transcribed into complementary DNA (cDNA) in a 10 μL reaction using the miRCURY LNA RT kit (Qiagen) according to the manufacturer’s recommendations. The process of RNA extraction and cDNA synthesis was monitored through synthetic spike-ins (RNA spike-in kit, Qiagen) added in both steps according to recommendations of the manufacturer.

### 4.3. Real-Time Quantitative Polymerase Chain Reaction (qPCR)

Based on the literature review, specific miRNAs were selected with potentially stable plasma expression (reference miRNAs, e.g., miR-16-5p, miR-103a-3p, miR-191-5p, miR-1228-3p, miR-520d-5p) and target miRNAs (miR-10b-5p, miR-99a-5p, miR-130a-3p, miR-342-3p, miR-484, miR-486-5p, miR-1260a) [10,17,52,60] with elevated expression in the plasma of cancer patients. The expression level of selected target and reference miRNAs was determined by the miRCURY LNA miRNA PCR system (Qiagen), and the quality of samples was tested by synthetic spike-in oligonucleotides (RNA Spike-in kit, Qiagen). All protocols for miRNA quantification were performed using a Bravo liquid handling station (Agilent, Santa Clara, CA, USA) and run on a LightCycler 480 instrument (Roche Diagnostics GmbH, Mannheim, Germany) with rapid analysis on LC480 instrument software by the second derivative method for Cq calculation. Secondary analysis of Cq values was performed in the GeneGlobe web tool (Qiagen), where isolation and transcription quality controls were evaluated, a stabilisation factor for reference miRNAs was calculated and data were normalised. These factors were taken into account when calculating the change in expression between the control group and the group of breast cancer patients with the corresponding *p*-value.

### 4.4. Statistical Analysis and Bioinformatics

miRNA expression and fold change (FC) were computed using the standard formula [61]. The data were visualised by a boxplot overlaid with a swarmplot. The null hypothesis that the population median FC is equal to 1 was tested by the Wilcoxon test. The null hypothesis that the population median FC is the same for two levels of a categorical clinical parameter (e.g., ER) was tested by the Wilcoxon two-sample test. The analogous null hypothesis for a categorical clinical parameter with more than two levels (e.g., grade), was tested by robust ANOVA. Findings with a *p*-value below 0.05 were considered statistically significant. The data analyses were performed in *R* [62] ver. 3.5.2, using the libraries beeswarm [63], robustbase [64] and the WRS2 package [65].

### 4.5. Signalling and Functional Analysis of Differentially Expressed miRNAs

The most statistically significant miRNAs were analysed using online software to assess the miRNA regulatory roles and identify controlled pathways (mirPath v.3) from the Kyoto Encyclopaedia of Genes and Genomes (KEGG) based on the functional pathway enrichment analysis, as well as multiple segments of Gene Ontology analysis (GO) in *Homo sapiens* [66]. The specific analysis tool was used to carry out the combination of the available in silico predicted targets from DIANA-micro T-CDS with high quality experimentally supported interactions. A *p*-value of <0.05 was considered statistically significant, and the Micro T threshold was set at 0.8. In the KEGG analysis, we prefer the gene union and in GO analysis, the category union and intersection for merging the results with false discovery rate (FDR) correction.

## Figures and Tables

**Table 1:** Clinicopathological data of the included patients.

Characteristics		Number of Patients (%)
All patients		65 (100%)
Age	Median (IQR)	56 (47,68)
	Missing	5
Histology	Ductal	21 (40%)
	Lobular	8 (15%)
	Invasive cancer, NST	24 (45%)
	Missing	12
Subtype	Luminal A	33 (63%)
	Luminal B	13 (25%)
	HER2	4 (7.7%)
	TNC	2 (3.8%)
	Missing	13
Grade	1	8 (14%)
	2	30 (53%)
	3	19 (33%)
	Missing	8
ER status	Positive	48 (87%)
	Negative	7 (13%)
	Missing	10
PR status	Positive	43 (78%)
	Negative	12 (22%)
	Missing	10
HER2	Positive	9 (18%)
	Negative	41 (82%)
	Missing	15
Tumour size	TIS	4 (6.9%)
	<20 mm	38 (66%)
	20 mm–50 mm	13 (22%)
	>50 mm	3 (5.2%)
	Missing	7
Nodal status	Negative	27 (56%)
	N1	17 (35%)
	N2	4 (8.3%)
	Missing	17
LVI	Positive	28 (51%)
	Negative	27 (49%)
	Missing	10
PNI	Positive	11 (20%)
	Negative	44 (80%)
	Missing	10
ki67	≤15%	34 (65%)
	>15%	18 (35%)
	Missing	13

Abbreviations: IQR, interquartile range; NST, no specific type; TNC, triple-negative cancer; ER, oestrogen; PR, progesterone; TIS, tumour in situ; mm, millimetre; LVI, lymphovascular invasion; PNI, perineural invasion.

**Table 2:** Fold change data analysis of tested circulating miRNAs in the plasma of cancer patients compared to the control group obtained in the Wilcoxon test. Significant values are displayed in bold.

miRNA ID	GeneGlobe Data Analysis Tool						ddCt Method		
	Pilot Testing			Final Testing					
	FC	Reg.	*p*-value	FC	Reg.	*p*-value	FC	Reg.	*p*-value
miR-10b	1.24	↑	0.17	0.86	↓	0.88	0.91	↓	0.68
miR-16	0.95	↓	0.45	0.95	↓	0.40	1	-	1
miR-99a	1.27	↑	0.22	1.06	↑	0.44	1.1	↑	0.03
miR-103a	1.06	↑	0.37	1.05	↑	0.62	1	-	1
miR-130a	0.94	↓	0.91	1.01	↑	0.34	1.33	↑	<0.01
miR-191	1.18	↑	0.13	-	-	-	-	-	-
miR-342	0.67	↓	0.05	0.76	↓	0.37	0.76	↓	0.35
miR-484	1.35	↑	0.01	1.14	↑	0.36	1.22	↑	<0.01
miR-486	0.90	↓	0.66	0.92	↓	0.34	0.89	↓	0.37
miR-520d	1.43	↑	0.31	-	-	-	-	-	-
miR-1228	1.43	↑	0.31	-	-	-	-	-	-
miR-1260a	0.82	↓	0.58	1.03	↑	0.12	1.2	↑	<0.01

Arrows represent up- or downregulation in breast cancer (BC) cases when compared to controls. Abbreviations: ID, identification; ddCt, delta–delta cycle threshold; FC, fold change; Reg., regulation.

**Table 3:** Kyoto Encyclopaedia of Genes and Genomes (KEGG) analysis of the significantly up-regulated miRNAs in the plasma of BC patients.

KEGG Pathway	Pathway ID	*p*-Value	miRNAs	Target Genes
Prion diseases	hsa05020	3.17 × 10^−22^	miR-130a-3p	*PNRP*
Hippo signalling pathway	hsa04390	0.007	miR-130a-3p	*WNT2B, FRMD6, TGFB2, TGFBR2, BMPR2, PPP1CB*
			miR-484	*YAP1, WNT2B, WWC1, TP53BP2, CDH1, AXIN2, DLG2, TCF7, FGF1*
			miR-99a-5p	*FDZ5, FDZ8, SMAD7*
			miR-1260a	*DLG2*
Phosphatidylinositol signalling system	hsa04070	0.0082	miR-130a-3p	*CDS1, DGKE, CALM2, PLCB1, PIKFYVE, PIK3C2A, PTEN, PLCB4, DGKH*
			miR-484	*CALM1, PLCZ1, PIKFYVE, PIK3CD*
			miR-1260a	*PIP4K2C, DGKH*
Oestrogen signalling pathway	hsa04915	0.0136	miR-484	*CREB3L3, CALM1, PIK3CD, GRM1, KCNJ5*
			miR-130a-3p	*ESR1, ADCY1, SOS2, CALM2, PLCB1, KCNJ6, HSPA8, PLCB4*
			miR-1260a	*ADCY1, ATF6B, KCNJ6, SP1*
Glioma	hsa05214	0.0136	miR-484	*CALM1, PIK3CD, PDGFA*
			miR-130a-3p	*SOS2, E2F2, TGFA, CALM2, IGF1, CDKN1A, PTEN*
			miR-99a-5p	*MTOR*
TGF-beta signalling pathway	hsa04350	0.0389	miR-484	*ACVR1B, PITX2*
			miR-130a-3p	*INHBB, SMURF2, INHBA, ACVR1, SKP1, ZFYVE9, SMAD5, TGFB2, TGFBR2, BMPR2*
			miR-1260a	*SKP1, SP1*
			miR-99a-5p	*SMAD7*
Glycosaminoglycan biosynthesis—heparan sulphate/heparin	hsa00534	0.0399	miR-1260a	*EXT2, HS3ST2*
			miR-99a-5p	*HS3ST3B1, HS3ST2*
mTOR signalling pathway	hsa04150	0.0399	miR-99a-5p	*MTOR*
			miR-130a-3p	*TSC1, RRAGD, PRKAA2, PRKAA1, IGF1, EIF4E2, PTEN, ULK2*
			miR-484	*RPS6KA1, PIK3CD*

**Table 4:** Gene Ontology (GO) annotation results of the target genes of up-regulated microRNAs in breast cancer samples.

GO Term	*p*-Value	Target Gene Account
**Biological process**		
GO:0034641: cellular nitrogen compound metabolic process	1.11 × 10^−16^	387
GO:0009058: biosynthetic process	4.86 × 10^−14^	241
GO:0048011: neurotrophin TRK receptor signalling pathway	8.09 × 10^−6^	31
GO:0006464: cellular protein modification process	1.64 × 10^−5^	151
GO:0007173: epidermal growth factor receptor signalling pathway	0.0002	17
GO:0006351: transcription, DNA-templated	0.0007	154
GO:0038095: Fc-epsilon receptor signalling pathway	0.0015	20
GO:0010467: gene expression	0.0035	25
GO:0007267: cell-cell signalling	0.0188	32
GO:0009056: catabolic process	0.0250	64
GO:0048015: phosphatidylinositol-mediated signalling	0.0315	10
GO:0034330: cell junction organisation	0.0471	11
GO:0008543: fibroblast growth factor receptor signalling pathway	0.0497	11
**Cellular component**		
GO:0043226: organelle	<1 × 10^−325^	772
GO:0043234: protein complex	3.68 × 10^−6^	239
GO:0005575: cellular component	0.00018	496
GO:0005829: cytosol	0.00667	96
GO:0005654: nucleoplasm	0.00765	47
**Molecular function**		
GO:0043167: ion binding	<1 × 10^−325^	Top of Form 488 Bottom of Form
GO:0003674: molecular function	1.374 × 10^−7^	Top of Form 515 Bottom of Form
GO:0001071: nucleic acid binding transcription factor activity	3.503 × 10^−6^	Top of Form 74 Bottom of Form
GO:0000988: protein binding transcription factor activity	1.861 × 10^−5^	Top of Form 50 Bottom of Form
GO:0019899: enzyme binding	5.208 × 10^−5^	Top of Form 98 Bottom of Form

## Supplementary Materials

Supplementary materials can be found at https://www.mdpi.com/1422-0067/21/19/7288/s1.
