# Supplementary material for: Pathway Analysis of Selected Circulating miRNAs in Plasma of Breast Cancer Patients: A Preliminary Study
Source: Int J Mol Sci. 2020 Oct 2;21(19):7288. doi: 10.3390/ijms21197288 (PMC7583045; doi:10.3390/ijms21197288)
Supplement: Supplementary file 1 [file ijms-21-07288-s001.pdf]

Supplementary Data

1. miRNA Expression Correlation to Histopathological Parameters

The causality of all miRNAs and histopathological parameters was tested by robust ANOVA and findings with the $p < 0.05$ were considered statistically significant. The summary of the ANOVA data analysis is listed in Table 1. The significant association was found between histology and miR-130a ($p = 0.005$) and tumor size and miR-99a ($p = 0.047$).

Table S1. Data analysis of circulating miRNA expression in the plasma of cancer patients, compared to control group according to histopathological parameters, the *p*-values were obtained in the Robust ANOVA method. The significant and weakly significant values are marked with asterisks.

miRNA ID	Histology	Subtype	Grade	ER Status	PR Status	HER2	Tumor Size	Nodal Status	LVI	PNI	ki67
**miR-10b-5p**	0.294	0.5	0.973	0.75	0.065*	0.911	0.17	0.743	0.561	0.127	0.901
**miR-99a-5p**	0.416	0.388	0.499	0.75	0.45	0.639	0.047*	0.728	0.506	0.782	0.319
**miR-130a-3p**	0.005*	0.277	0.711	0.606	0.43	0.961	0.749	0.92	0.142	0.554	0.971
**miR-144-3p**	0.815	0.855	0.78	0.589	0.823	0.971	0.457	0.395	0.352	0.353	0.503
**miR-342-3p**	0.655	0.39	0.398	0.777	0.323	0.088*	0.691	0.743	0.635	0.705	0.31
**miR-451**	0.812	0.583	0.944	0.84	0.897	0.591	0.414	0.817	0.861	0.539	0.807
**miR-484**	0.442	0.819	0.519	0.695	0.262	0.757	0.301	0.591	0.315	0.582	0.904
**miR-486-5p**	0.654	0.583	0.711	0.623	0.256	0.388	0.215	0.085*	0.546	0.863	0.518
**miR-1260a**	0.771	0.382	0.94	0.952	0.655	0.983	0.482	0.806	0.298	0.49	0.614

MicroRNA-99a was significantly associated with tumor size ($p = 0.047$). However, the main difference is between tumor in situ (TIS) and tumor size less than 20 mm. In the group of TIS, there is a small samples size ($n = 4$) and the results are not representative (Figure 1). MicroRNA-130a was found significantly associated with histology type ($p = 0.005$). After detailed observation of the dotplot, the main difference is between invasive lobular carcinoma (ILC) and invasive carcinoma—no specific type (IC NST) that can be hardly specified (Figure 1).
